# Identification and diagnostic significance of MPO, PRTN3, and CTNND1 as biomarkers in acute hematogenous osteomyelitis in children: a comprehensive analysis using machine learning algorithms

**DOI:** 10.3389/fped.2025.1565619

**Published:** 2025-11-07

**Authors:** Xin Lv, Jiafei Yang, Kezhi Chen, Jihang Luo, Tianjiu Zhang, Song Yu

**Affiliations:** 1 Department of Pediatric Surgery, Affiliated Hospital of Zunyi Medical University, Zunyi, China; 2 Department of Pediatric Surgey, Guizhou Children's Hospital, Zunyi, China; 3 Department of Pediatric Surgery, The Affiliated Hospital of Guizhou Medical University, Guizhou Medical University, Guiyang, China

**Keywords:** acute hematogenous osteomyelitis, biomarkers, CTNND1, PRTN1, MPO

## Abstract

**Introduction:**

Acute hematogenous osteomyelitis (AHO) is a severe bacterial bone infection predominantly affecting children. Early diagnosis is crucial to prevent the progression to chronic osteomyelitis. However, current diagnostic methods are limited in sensitivity and specificity, underscoring the need for reliable biomarkers.

**Materials and methods:**

This study utilized gene expression data from the Gene Expression Omnibus (GEO) to identify differentially expressed genes (DEGs) associated with AHO. We employed three machine learning algorithms—The Least Absolute Shrinkage and Selection Operator (LASSO) regression, Support Vector Machine-Recursive Feature Elimination (SVM-RFE), and Random Forest (RF)—to screen for potential diagnostic markers. The expression levels of key genes were validated using clinical samples from pediatric AHO patients. Receiver operating characteristic (ROC) curve analysis was performed to assess the diagnostic accuracy of these biomarkers.

**Results:**

Our analysis identified five candidate genes, among which Myeloperoxidase (MPO), Serum proteinase 3 (PRTN3), Catenin delta 1 (CTNND1) were significantly associated with AHO, MPO and PRTN3 were upregulated, while CTNND1 was downregulated in AHO samples compared to healthy controls. ROC curve analysis demonstrated that CTNND1 (AUC = 0.8832), MPO (AUC = 0.9803) and PRTN3 (AUC = 0.9767) exhibited strong diagnostic potential. Importantly, the expression levels of MPO and PRTN3 positively correlated with disease severity as classified by the Cierny-Mader staging system, whereas CTNND1 expression showed a negative correlation.

**Conclusion:**

MPO, PRTN3, and CTNND1 are promising biomarkers for the diagnosis and monitoring of AHO in children. Their expression levels correlate with disease severity, making them valuable tools for assessing the progression and treatment efficacy in pediatric AHO. Further research is warranted to explore their potential in clinical applications.

## Introduction

Acute hematogenous osteomyelitis (AHO) is a bacterial bone infection that arises from hematogenous spread, primarily affecting young children (1), In high-income countries, the incidence is approximately 8 per 100,000 children annually (1, 2). AHO is the most common form of osteomyelitis in pediatric patients and often necessitates hospitalization, invasive diagnostic procedures, surgical intervention, and prolonged antimicrobial therapy (3). Staphylococcus aureus (S. aureus) is the predominant pathogen, with both methicillin-sensitive Staphylococcus aureus and methicillin-resistant Staphylococcus aureus being frequently implicated (4). Current treatment strategies emphasize antimicrobial therapy and meticulous debridement to manage these infections. However, as the disease progresses, complications such as antibiotic resistance and abscess formation can significantly compromise treatment efficacy (5). Thus, early diagnosis and timely intervention to prevent the transition from acute to chronic osteomyelitis are critical.

The clinical diagnosis of acute osteomyelitis typically involves a multifaceted approach, including the evaluation of clinical signs, radiological imaging, and laboratory test results. Bone biopsy and microbiological culture are often employed to confirm the diagnosis (6). Although x-rays are commonly the initial imaging modality, their sensitivity in detecting early osteomyelitis is limited, diminishing their diagnostic utility. In pediatric osteomyelitis, radiographic changes typically become apparent around two weeks after disease onset, whereas in adults, these changes may take longer to manifest. Magnetic resonance imaging is considered the gold standard for osteomyelitis imaging due to its high sensitivity in detecting early-stage lesions. However, its high cost and limited impact on treatment outcomes present significant challenges (7). The lack of rapid, direct, and specific diagnostic tools creates substantial obstacles in the timely and accurate diagnosis of osteomyelitis, highlighting the urgent need for the identification of suitable non-invasive biomarkers in AHO patients.

With the advancement of high-throughput sequencing technologies, high-throughput genetic microarray analysis of various sample types has enabled the investigation of diseases at multiple levels, including DNA, RNA, and protein. Recent studies have identified numerous specific genes involved in the progression of osteomyelitis. In a recent study involving a Chinese population, a comparison of gene polymorphisms between cases of post-traumatic osteomyelitis and healthy controls revealed that polymorphisms in the NLR family pyrin domain containing 3, elongator complex protein 2, signal transducer and activator of transcription 3, caspase 1, nuclear factor of kappa light polypeptide gene enhancer in B-cells inhibitor, alpha, nuclear factor of kappa light polypeptide gene enhancer in B-cells 1, caspase recruitment domain family member 8, and cluster of differentiation 14 genes are associated with an increased risk of post-traumatic osteomyelitis. Specifically, variants rs10754558 and rs7525979 of the NLRP3 gene were found to significantly increase the risk of developing post-traumatic osteomyelitis (8). Further cellular studies demonstrated that S. aureus induces the activation of the NLRP3 inflammasome, and inhibition of NLRP3 inflammasome activity by MCC950 reduces S. aureus-induced bone resorption and the expression of osteoclast-specific genes, including tartrate-resistant acid phosphatase, matrix metallopeptidase 9 (MMP9), cathepsin K, calcitonin receptor, and vacuolar ATPase V0 subunit d2 (9). Additionally, Campbell et al. revealed that S. aureus significantly upregulates the expression of the receptor activator of nuclear factor kappa-*Β* ligand (RANKL) gene, and treatment with denosumab completely prevents severe cortical bone destruction caused by the infection, indicating that RANKL-mediated osteoclastogenesis is essential for bone loss induced by S. aureus infection (10). These findings suggest the critical role of certain functional genes in the progression of osteomyelitis. However, the diagnostic value of many genes in osteomyelitis remains to be explored.

In this study, we utilized data from the Gene Expression Omnibus (GEO) to identify differentially expressed genes (DEGs) associated with AHO. We constructed a nomogram model to evaluate the diagnostic potential of key genes in osteomyelitis and assessed the model's diagnostic accuracy through receiver operating characteristic (ROC) curve analysis. The reliability of the model was further validated using an independent dataset.

## Materials and methods

### Data preprocessing

The gene expression dataset GSE30119 and its corresponding platform annotation files were obtained from the GEO database. We selected this dataset because it (i) focuses on pediatric Staphylococcus aureus infections, (ii) contains healthy controls, and (iii) provides clear metadata to identify bone/joint–only cases. From this dataset, we included 12 pediatric samples with infections confined to bone and joint and 22 healthy controls. Samples with multiple infection sites or incomplete metadata were excluded.

Raw data were background-corrected, log2-transformed, and normalized using the limma package in R. Probe IDs were mapped to official gene symbols based on the platform annotation; if multiple probes mapped to the same gene, the probe with the highest average expression was retained. To minimize potential technical variation, we inspected principal components before and after adjustment and applied the ComBat function from the sva package for batch-effect correction, with group information (AHO vs. control) preserved. Post-correction PCA confirmed reduced batch-associated variation while maintaining biological separation. The processed dataset was then used for downstream differential expression and machine learning analyses.

### Identification of DEGs

Differential expression analysis was conducted on 48,003 AHO-related genes using the “limma” package to identify differentially expressed genes between osteomyelitis samples and healthy controls. Visualizations were generated using the “Matplotlib” and “pheatmap” packages, resulting in a volcano plot and a heatmap, respectively. The differentially expressed genes were then further filtered based on an adjusted *p*-value of less than 0.05 and a log-fold change (logFC) greater than |1|.

### Functional enrichment analysis

The potential biological functions of the differentially expressed AHO genes were investigated through enrichment analysis. This analysis included Gene Ontology (GO) categories—biological process (BP), cellular component (CC), and molecular function (MF)—as well as Kyoto Encyclopedia of Genes and Genomes (KEGG) pathways. The enrichment analysis was performed using the online platform Metascape (https://metascape.org). The top five significantly enriched functions and pathways were identified and visualized in the form of enrichment network diagrams, with a *P*-value of less than 0.05 considered indicative of significant enrichment.

### Candidate diagnosis marker selection

Three machine learning algorithms were applied to identify potential diagnostic factors and predict AHO status. The Least Absolute Shrinkage and Selection Operator (LASSO), a regression analysis algorithm, was employed to improve prediction accuracy through regularization. LASSO regression analysis was conducted using the “glmnet” library in Python to identify genes significantly associated with the discriminative power between AHO and healthy samples. The Support Vector Machine (SVM), a supervised machine learning technique widely used for classification and regression analysis, was also utilized. To avoid overfitting, the Recursive Feature Elimination (RFE) algorithm was applied to select the optimal genes from the metadata cohort, with SVM-RFE being used specifically to identify features with the greatest discriminatory ability. Additionally, the Random Forest (RF) algorithm, an ensemble learning method based on decision trees, was implemented using the “randomForest” library in Python to enhance classification and prediction accuracy by constructing multiple decision trees. This algorithm was further employed to filter key genes associated with AHO status.

### Patient and ethics statement

The participants in this study were AHO patients treated at the Affiliated Hospital of Zunyi Medical University between July 2022 and July 2024 (*n* = 61). The healthy control group consisted of children undergoing routine physical examinations, excluding those with infectious diseases. AHO diagnosis was based on clinical symptoms (localized bone pain, fever, swelling, restricted mobility), laboratory markers (elevated WBC, CRP, ESR, PCT), and MRI findings; in doubtful cases, bone aspiration or surgical specimens were cultured. Diagnostic criteria followed the Pediatric Infectious Diseases Society and Infectious Diseases Society of America guidelines (2).

The study protocol was approved by the Ethics Committee of the Affiliated Hospital of Zunyi Medical University (No. KL2022-450). All procedures complied with institutional standards and the Declaration of Helsinki. Written informed consent was obtained from all participants.

### Biochemical assessment

Peripheral blood was collected from each patient before any AHO treatment and on the third day post-operation. Serum was obtained by centrifugation at 3,000 rpm for 5 min and stored at −80°C until testing. All serum samples were stored in duplicate. White blood cell (WBC) counts were measured using an automated blood analyzer. C-reactive protein (CRP) levels were determined by immunoturbidimetry. The erythrocyte sedimentation rate (ESR) was measured using the Westergren method, and procalcitonin (PCT) levels were assessed by electrochemiluminescence immunoassay. MPO levels were determined using a time-resolved fluorescence lateral flow immunoassay based on the principle of immunochromatography and a double antibody sandwich method (Eachy Biopharma, China). Serum PRTN3 concentrations were measured using a human PRTN3 enzyme-linked immunosorbent assay (ELISA) kit (ab226902; Abcam, Tokyo, Japan) according to the manufacturer's instructions. Serum CTNND1 concentrations were measured using an ELISA kit (SEE901Mu; Cloud-Clone Corp, Wuhan, China) according to the manufacturer's instructions.

### Statistical analysis

All experimental data were analyzed using SPSS software (version 22.0; IBM, Armonk, NY, USA) and GraphPad Prism 5.0 (GraphPad Software, La Jolla, CA, USA). To evaluate the classification performance of key genes in AHO and healthy samples, ROC curves were generated to assess their diagnostic value. A *P*-value of less than 0.05 was considered statistically significant.

## Result

### Screening for diferentially expressed AHO-associated genes

**Figure 1:**
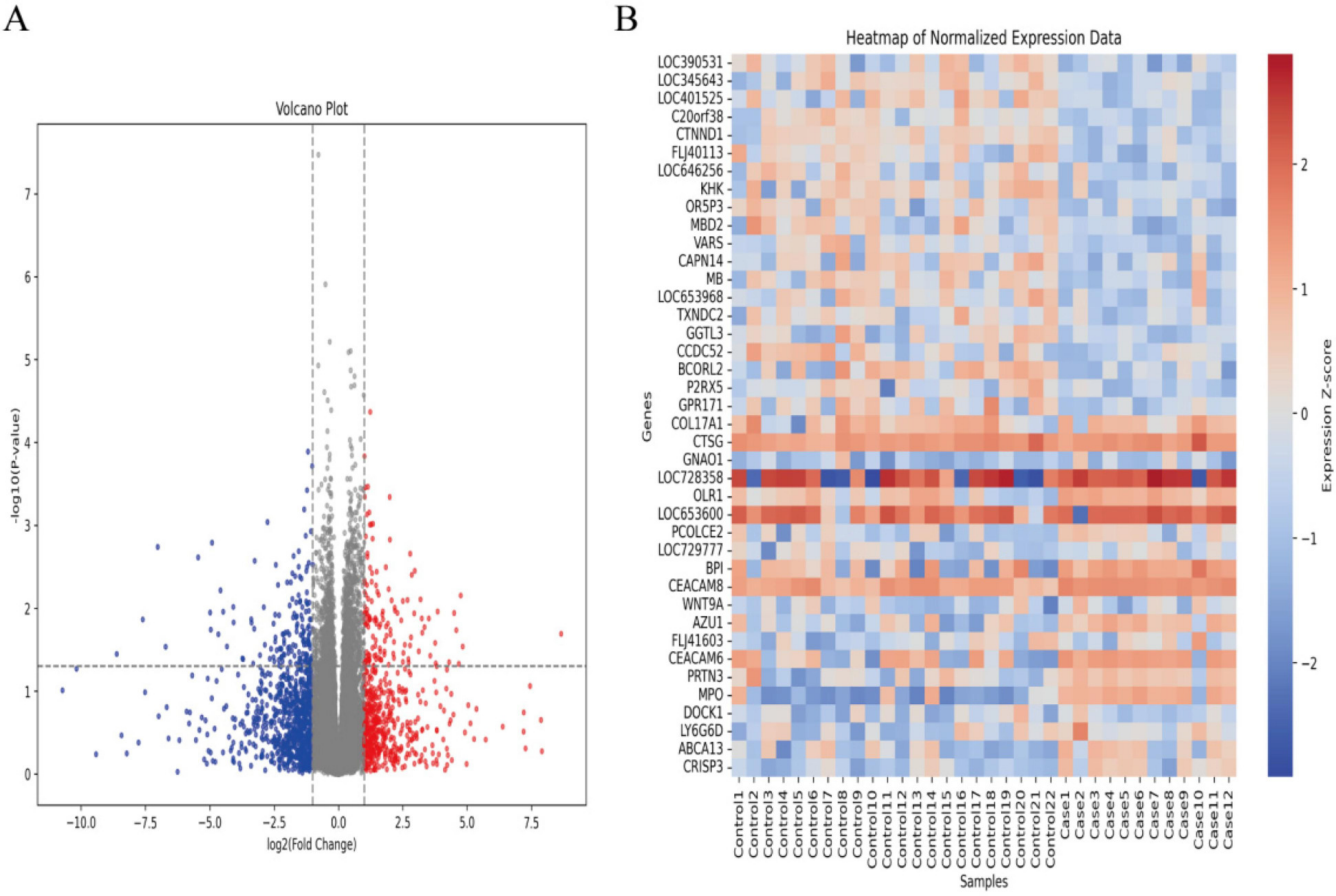
Identifcation of diferentially expressed genes (DEGs) in AHO and normal groups. **(A)** Volcano plot of DEGs in samples from AHO and controls in GSE30119. Red dots represent up-regulated genes and blue dots represent down-regulated genes in AHO samples, gray indicates no signifcance. **(B)** Heatmap for DEGs in HCM (red) and controls (green).

In the GSE30119 dataset, after screening, 22 healthy controls and 12 cases of osteomyelitis or pyogenic arthritis without other comorbidities were identified. Differential expression analysis between these two groups was conducted to identify potential biomarkers for AHO. A total of 279 genes were found to be significantly differentially expressed between the normal and AHO groups (|LogFC| > 1), with 125 genes upregulated and 154 genes downregulated in AHO. The corresponding volcano plot and heatmap are shown in Figures 1A,B, respectively.

### Functional enrichment analyses

To elucidate the biological functions of these regulators in AHO, we conducted GO enrichment analysis and KEGG pathway analysis using the Bioconductor package clusterProfiler in R. To better distinguish the biological functions of upregulated and downregulated genes, GO and KEGG analyses were performed separately for the upregulated and downregulated gene sets.

**Figure 2:**
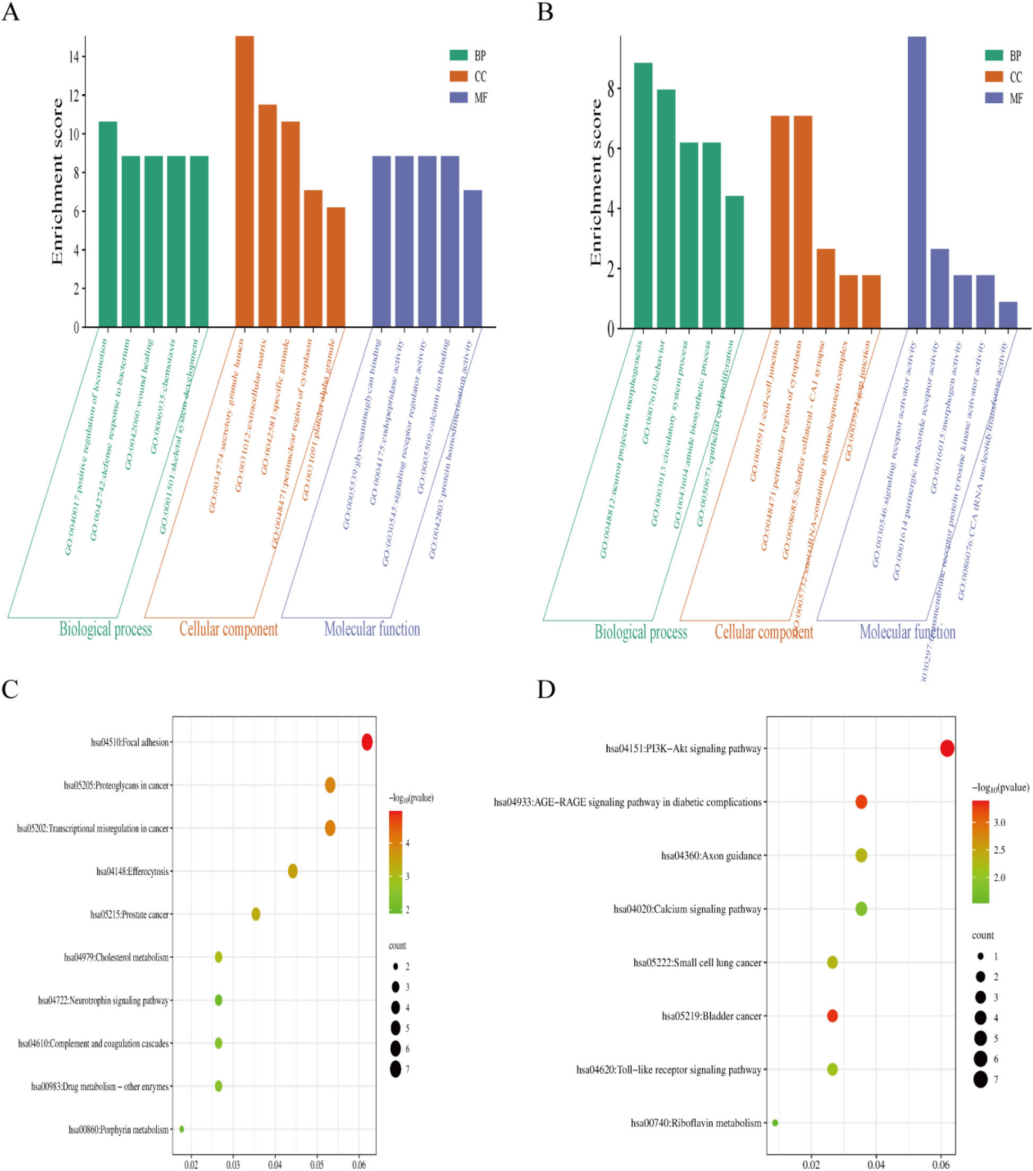
The gene ontology(GO) and Kyoto Encyclopedia of genes and genomes (KEGG) enrichment analyses using the |LogFC| > 1related DEGs. **(A,C)** GO and KEGG enrichment analyses shows enriched items in the Log FC > 1 related DEGs. **(B,D)** GO and KEGG enrichment analyses shows enriched items in the Log FC < −1 related DEGs. BP, biological process; CC, cellular component; MF, molecular function.

First, we analyzed the upregulated genes. The GO enrichment analysis revealed that, in terms of BP, AHO was predominantly associated with positive regulation of locomotion, defense response to bacterium, and wound healing. For CC, these genes were mainly involved in the secretory granule lumen and specific granule. Regarding MF, the upregulated genes were primarily linked to glycosaminoglycan binding and endopeptidase activity (Figure 2A). Pathway analysis (Figure 2C) showed significant enrichment in the Focal adhesion, Transcriptional misregulation in cancer, and Proteoglycans in cancer pathways.

Next, we analyzed the downregulated genes. The GO enrichment analysis indicated that, in terms of BP, AHO was associated with neuron projection morphogenesis, behavior, and circulatory system processes. For CC, these genes were involved in the cell-cell junction, perinuclear region of the cytoplasm, and Schaffer collateral-CA1 synapse. Regarding MF, the downregulated genes were primarily linked to signaling receptor activator activity, purinergic nucleotide receptor activity, and morphogen activity (Figure 2B). Pathway analysis (Figure 2D) revealed significant enrichment in the PI3K-Akt signaling pathway, Calcium signaling pathway, and AGE-RAGE signaling pathway in diabetic complications.

### Screening target genes by LASSO regression, SVM-RFE and RF analysis

**Figure 3:**
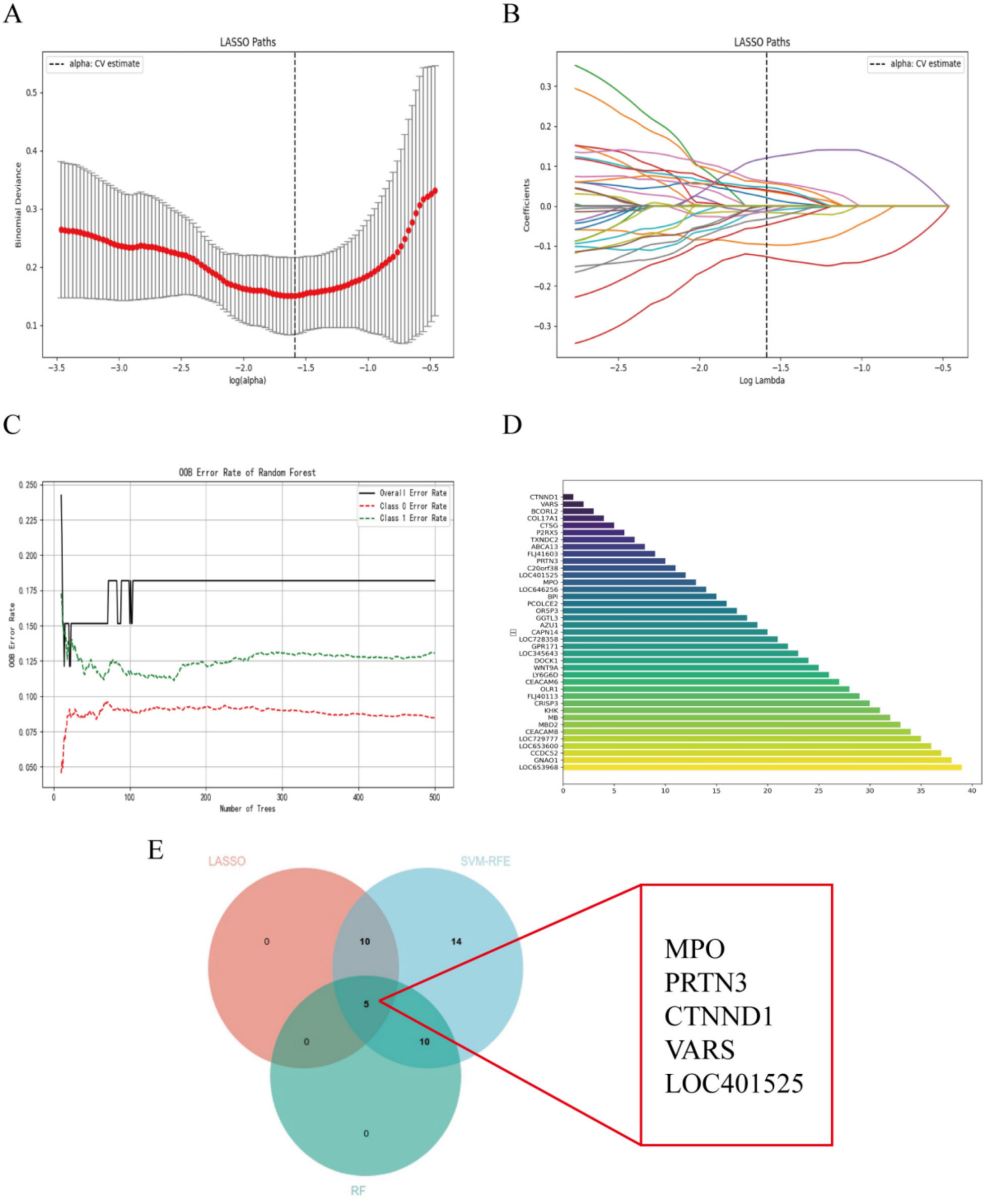
Selection of diagnosis marker candidates for AHO: **(A,B)** tuning feature screening in the LASSO model; **(C)** RF coefficient profiles of candidate genes; **(D)** a plot of biological markerscreening via the SVM-RFE arithmetic; **(E)** venn graph displaying 5 diagnosis biomarkers shared by LASSO, SVM-RFE and RF.

The DEGs were identified using the LASSO regression algorithm, which resulted in the identification of 15 variables as diagnostic markers for AHO (Figures 3A,B). Additionally, we identified 15 key genes using the RF algorithm (Figure 3C). A subset of 39 features among the DEGs was identified using the SVM-RFE algorithm (Figure 3D). The three overlapping features (MPO, PRTN3, CTNND1, VARS, LOC401525) among these three algorithms were ultimately selected (Figure 3E). These five genes may be critical in the progression of AHO.

### The expression and diagnosis significance of candidate genes in AHO

**Figure 4:**
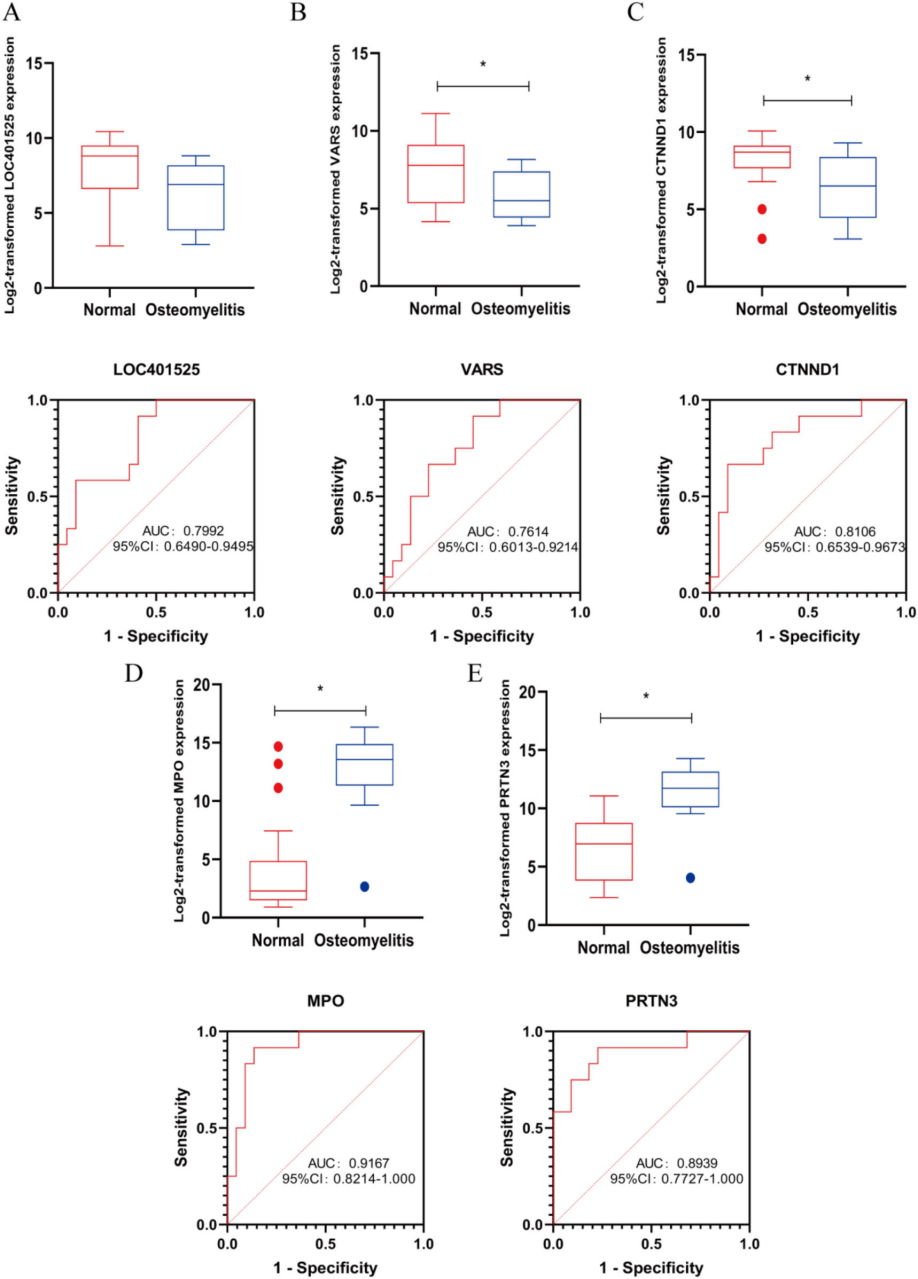
Marker candidates in sequencing samples from patients with AHO vs. healthy controls. **(A–E)** LOC401525, VARS, CTNND1, MPO and PRTN3 expression levels in GSE30119. Receiver operating characteristic (ROC) curve for LOC401525, VARS, CTNND1, MPO and PRTN3 in GSE30119 samples from AHO patients. * <0.05.

Our team found that there was no statistically significant difference in the expression level of LOC401525 between the control and experimental groups (*P* > 0.05). The expression levels of Variance Adjusted Response Selection (VARS) and CTNND1 were significantly downregulated in AHO samples compared to healthy samples (*P* < 0.05) (Figures 4A–C), while the expression levels of MPO and PRTN3 were significantly upregulated in AHO samples (*P* < 0.05) (Figures 4D,E). To further explore the diagnostic value of these candidate genes, we performed ROC curve analyses. The results showed that VARS (Figure 4B, AUC = 0.761), CTNND1 (Figure 4C, AUC = 0.811), MPO (Figure 4D, AUC = 0.917), and PRTN3 (Figure 4E, AUC = 0.894) exhibited varying degrees of diagnostic ability in distinguishing AHO samples from normal samples.

### General characteristics of the study population

A total of 61 patients with AHO and 25 healthy patients were recruited into the study. The demographic, clinical, and biochemical characteristics are provided in Table 1. There were no significant differences between the two groups regarding sex, age, height, or weight (*p* > 0.05 for all). However, in the AHO group, the levels of WBC, CRP, ESR, and PCT were significantly higher compared to the healthy control group (*p* < 0.05). According to the Cierny-Mader staging system, AHO is classified into four stages. However, due to the rarity of Stage IV cases, they were not included in the statistical analysis of this study. As shown in Table 2, there were no significant statistical differences in age, gender, height, or weight (*p* > 0.05). However, WBC, CRP, ESR, and PCT levels showed statistically significant differences, indicating that these indicators are related to the severity of the disease.

**Table 1:** Baseline characteristics and factors of all the patients included in the study.

Demographic data	Healthy	Osteomyelitis	*p*-value
Age (years)	4.8 ± 2.1	3.8 ± 2.2	0.067
Sex (males, *n*)	14 (56%)	39 (64%)	0.500
Height (cm)	106.2 ± 13.9	100.4 ± 18.3	0.154
Weight (Kg)	18.4 ± 6.1	15.9 ± 5.6	0.071
WBC (/ul)	8,416.0 ± 1,820.0	16,027 ± 6,435.0	<**0**.**001**
CRP (mg/l)	4.0 ± 1.6	69.0 ± 43.2	<**0**.**001**
ESR (mm/h)	6.8 ± 3.2	60.6 ± 42.0	<**0**.**001**
PCT (ng/ml)	0.03 ± 0.01	1.65 ± 2.12	<**0**.**001**

Values are presented as mean ± SD or number (percentage). Bold values indicate statistically significant differences (*p* < 0.001).

**Table 2:** Characteristics of AHO patients based on the Cierny-Mader staging system.

Demographic data	Stage I	Stage II	Stage III	*p*-value
Age (years)	3.8 ± 2.2	4.0 ± 2.2	3.6 ± 2.3	0.879
Sex (males, *n*)	19 (65%)	17（59%）	17 (67%)	0.878
Height (cm)	99.8 ± 19.0	101.9 ± 18.0	99.8 ± 18.4	0.920
Weight (Kg)	15.1 ± 5.4	16.6 ± 5.3	16.5 ± 6.5	0.598
WBC (/ul)	10,737.9 ± 2,061.1	17,500.0 ± 4,548.4	24,586.7 ± 2,499.1	<**0**.**001**
CRP (mg/l)	29.8 ± 13.2	62.3 ± 15.3	113.3 ± 23.3	<**0**.**001**
ESR (mm/h)	23.9 ± 14.7	84.6 ± 21.7	112.3 ± 18.9	<**0**.**001**
PCT (ng/ml)	0.21 ± 0.26	2.07 ± 1.85	3.94 ± 2.24	<**0**.**001**

Values are presented as mean ± SD or number (percentage). Bold values indicate statistically significant differences (*p* < 0.001).

### MPO, PRTN3 and CTNND1 expression level in peripheral blood cells is an independent predictive biomarker for AHO diagnosis

**Figure 5:**
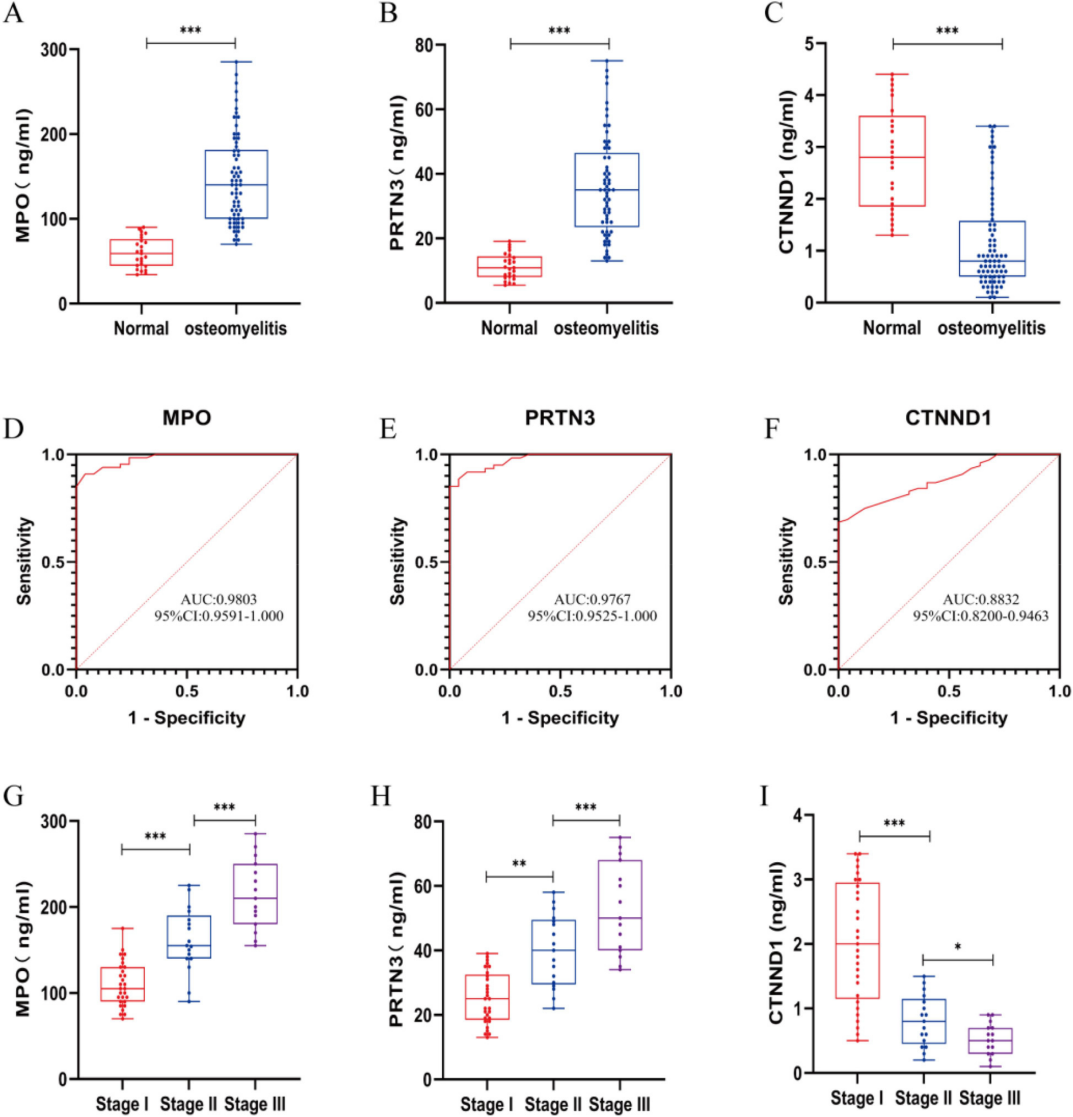
Inflammatory markers, MPO, PRTN3 and CTNND1 concentrations and ROC in plasma samples from AHO vs. healthy cantrol. **(A–C)** Expression for MPO, PRTN3 and CTNND1 concentrations in plasma samples from patients with AHO (*n* = 61) vs. healthy control (*n* = 25). **(D–F)** ROC curves for MPO, PRTN3 and CTNND1 from patients with AHO (*n* = 61) vs. healthy control (*n* = 25). **(G–I)** According to the Cierny-Mader staging system, patients with AHO at different stages are classified into Stage I, Stage II, and Stage III to compare the expression levels of MPO, PRTN3, and CTNND1 in plasma.*p < 0.05, **p < 0.01, ***p < 0.001 by unpaired Student's *t*-test.

We performed clinical tests on peripheral blood from AHO patients and healthy individuals and found that MPO, PRTN3, and CTNND1 exhibited significant statistical differences, with *p*-values less than 0.05 (Figures 5A–C). These indicators were significantly elevated in AHO patients. To further validate the diagnostic efficiency of MPO, PRTN3, and CTNND1 for AHO patients, we plotted ROC curves, where the AUC for MPO, PRTN3, and CTNND1 was 0.9803, 0.9767, and 0.8832, respectively (Figures 5D–F). According to the Cierny-Mader staging system, we classified osteomyelitis into three stages. MPO and PRTN3 levels in serum increased with the progression of the stages, showing statistically significant differences between each stage. In contrast, CTNND1 levels in serum decreased as the stage advanced, with statistically significant differences observed between Stage I, Stage II, and Stage III (Figures 5G–I).

**Figure 6:**
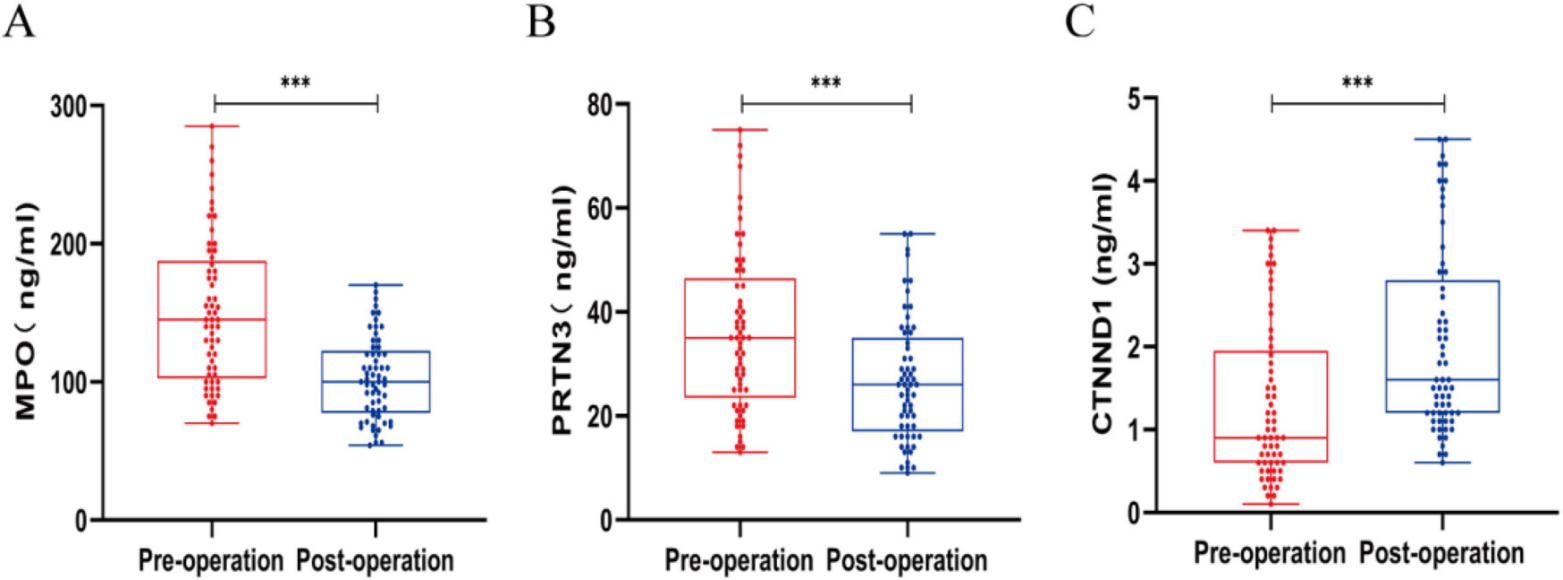
Expression levels of MPO **(A)**, PRTN3 **(B)**, and CTNND1 **(C)** in peripheral blood cells before and after surgery in patients with acute hematogenous osteomyelitis (AHO). Data are shown as mean ± SD. *p < 0.05, **p < 0.01, ***p < 0.001 by unpaired Student's t-test.

Next, we evaluated the expression levels of MPO, PRTN3, and CTNND1 in peripheral blood cells obtained from AHO patients before and after surgery. Interestingly, the expression levels of MPO and PRTN3 in peripheral blood were significantly lower in the postoperative state compared to the preoperative state. In contrast, the expression level of CTNND1 was significantly higher after surgery (*P* < 0.05; Figures 6A–C).

## Discussion

AHO is the most common cause of osteomyelitis in children (11). Without timely treatment, it may progress to chronic disease and severely impair quality of life (12). Therefore, early and specific diagnostic methods remain urgently needed in clinical practice. Through integrated GEO analysis, we identified 279 DEGs (125 upregulated, 154 downregulated). Upregulated genes mapped to bacterial defense, wound healing, and ECM pathways, while downregulated genes were enriched in junctional and neuro-immune signaling, implicating PI3K–Akt and calcium pathways. These findings suggest that these genes are inhibited during inflammation and may play a crucial role in mitigating the progression of AHO.

Machine-learning–based feature selection yielded five candidates (LOC401525, VARS, CTNND1, MPO, PRTN3). Validation highlighted MPO, PRTN3, and CTNND1 as the most robust markers, while LOC401525 and VARS showed weaker performance. Among these, LOC401525, VARS, and CTNND1 were downregulated, while MPO and PRTN3 were upregulated. Currently, there are no literature reports on the function of LOC401525. VARS is a valyl-tRNA synthetase, primarily responsible for attaching valine to tRNA during protein synthesis (13). Current research suggests that VARS is mainly associated with hereditary neurological diseases (14). The CTNND1 gene encodes p120-catenin, a critical component of adherens junctions that preserves cell–cell adhesion and tissue integrity (15, 16). In osteomyelitis, disruption of these junctions facilitates bacterial invasion and spread, and reduced CTNND1 expression may therefore compromise structural barriers against Staphylococcus aureus. Beyond its adhesive function, accumulating evidence indicates that p120-catenin also acts as a regulator of the inflammatory microenvironment. Experimental studies have shown that loss of p120 enhances NF-κB signaling and increases epithelial and endothelial permeability, thereby promoting leukocyte infiltration and sustaining local inflammation (17, 18). Moreover, p120 interacts with Rho family GTPases to modulate cytoskeletal dynamics and inflammatory cell trafficking (19). In addition, p120 is functionally linked to canonical Wnt/β-catenin signaling, which is essential for maintaining osteoblast–osteoclast balance and bone homeostasis (17). Taken together, downregulation of CTNND1 in pediatric AHO may contribute not only to weakened cell adhesion and junctional stability but also to amplification of pro-inflammatory signaling and disruption of bone remodeling. These mechanisms provide a plausible explanation for our observation of reduced CTNND1 expression in AHO patients and highlight its potential value as both a diagnostic biomarker and a target for future mechanistic investigations.

Using machine learning algorithms, we identified LOC401525, VARS, and CTNND1 as downregulated genes in AHO samples. However, cohort studies using sequencing data revealed that LOC401525 had a *p*-value greater than 0.05, indicating no statistically significant difference. ROC analysis of the data for VARS and CTNND1 showed AUC values of 0.7614 and 0.8106, respectively. Given the current literature on VARS and the observation that the AUC curve for VARS was significantly lower than that of the other three genes during ROC analysis, further testing of VARS was not pursued in subsequent human experiments. Based on related research and our experimental studies, we believe that CTNND1 is a potential diagnostic biomarker for AHO.

MPO is a heme-containing enzyme primarily produced by neutrophils and monocytes, playing a critical role in immune responses and inflammation (20). It is predominantly located in the lysosomes of these cells. During an inflammatory response, neutrophils generate hydrogen peroxide through a respiratory burst, and MPO utilizes this hydrogen peroxide to react with chloride ions, producing hypochlorous acid. Hypochlorous acid is a potent oxidant capable of killing a wide range of pathogens, including bacteria, fungi, and viruses (20, 21). However, the hypochlorous acid and other oxidative products generated by MPO not only exert bactericidal effects but also have the potential to damage host cells and tissues (22). Current research indicates that MPO contributes to the pathogenesis of osteomyelitis not only through its antimicrobial activity but also by exacerbating local inflammatory responses via the production of oxidative products. These oxidative products can induce oxidative stress, leading to tissue damage, further intensifying inflammation, and consequently delaying the healing of bone tissue (22, 23). In chronic osteomyelitis, sustained MPO activity may result in a prolonged inflammatory state, hindering bone tissue repair, and ultimately leading to persistent tissue destruction and functional loss. Additionally, elevated MPO levels in osteomyelitis patients have been found to correlate with disease severity (24). PRTN3 is a serine protease primarily located in the granules of neutrophils. In the antibacterial defense mechanism of neutrophils, PRTN3 plays a crucial role by cleaving microbial proteins and directly killing pathogens (25). Moreover, PRTN3 can activate other antimicrobial molecules, further enhancing the bactericidal capacity of neutrophils (26). Additionally, PRTN3 is capable of cleaving and modulating various cytokines and chemokines, thus influencing the intensity and duration of the inflammatory response (22). However, overexpression of PRTN3 may disrupt the bone matrix and soft tissues, impeding bone tissue repair and reconstruction, thereby delaying the recovery process (27). In our cohort, MPO and PRTN3 were consistently elevated in AHO, correlated with severity, and declined after treatment, while CTNND1 showed the opposite trend. Together, these dynamics highlight their potential as diagnostic and monitoring biomarkers. Additionally, current literature suggests that a sustained inflammatory response involving MPO and PRTN3 may contribute to the transition from acute to chronic osteomyelitis. Further experiments could be conducted to explore the mechanisms of chronic osteomyelitis formation by regulating MPO and PRTN3.

Previous studies have extensively explored potential biomarkers of Staphylococcus aureus–induced OM using transcriptomic and bioinformatic approaches. For example, Chen et al. identified DEGs in OM from GSE30119 and demonstrated enrichment in immune-related pathways, highlighting the critical role of neutrophil extracellular traps and immune escape mechanisms (28). More recently, Shi et al. proposed ferroptosis-related signatures with excellent diagnostic capacity (AUC = 0.993), emphasizing ferroptosis and amino acid metabolism in OM pathogenesis (29). Similarly, other investigations have revealed cuproptosis-related gene signatures associated with M2 macrophage polarization, suggesting their value in early diagnosis and immune regulation of OM (30). Molecular subgroup analyses have further shown heterogeneity among OM patients, where distinct immune infiltration patterns and osteoclast-related pathways (e.g., CTSK) correlated with disease severity and length of hospital stay (31). Unlike previous bioinformatic-only analyses, our work validates biomarkers in a pediatric clinical cohort, links them to severity and treatment response, and emphasizes neutrophil granule biology and adhesion integrity as novel axes. Taken together, our results not only align with the growing consensus that immune-related genes are key diagnostic indicators in osteomyelitis, but also extend prior research by validating novel markers in a pediatric cohort, thereby providing potential clinical utility for early and accurate diagnosis of AHO. Future multicenter studies with larger populations will be necessary to compare the performance of these biomarkers across diverse subgroups and to explore their integration with ferroptosis- and cuproptosis-related signatures for improved disease stratification.

Compared with conventional inflammatory markers such as WBC, CRP, ESR, and PCT, which are sensitive but nonspecific, these new biomarkers exhibited superior diagnostic performance. MPO and PRTN3 achieved excellent AUC values (0.9803 and 0.9767, respectively), higher than those generally reported for conventional markers. Moreover, their expression levels dynamically correlated with Cierny-Mader stages and declined after treatment, indicating value not only in diagnosis but also in monitoring therapeutic efficacy. CTNND1, conversely, decreased with disease severity but increased after treatment, suggesting a protective role. These results indicate that MPO, PRTN3, and CTNND1 are complementary to conventional markers but provide greater specificity and dynamic monitoring capability for pediatric AHO.

In this study, we validated that MPO, PRTN3, and CTNND1 are differentially expressed in children with acute hematogenous osteomyelitis and exhibit strong diagnostic performance, with their serum levels correlating with disease severity and treatment response. These results confirm their potential utility as biomarkers for early diagnosis and disease monitoring in pediatric AHO. However, while our data suggest possible mechanistic links between CTNND1 and inflammatory signaling pathways, as well as a role for MPO and PRTN3 in the transition from acute to chronic disease, these interpretations remain speculative. Future studies, particularly large-scale multicenter and mechanistic investigations, are required to validate these hypotheses and to determine the translational value of these biomarkers in clinical practice.

### Limitations and perspectives

This study has several limitations that should be acknowledged: (1) Sample size – Only 61 pediatric AHO patients and 25 healthy controls were included. Although MPO, PRTN3, and CTNND1 showed significant differences and high diagnostic accuracy (AUC >0.88), the statistical power remains limited for detecting moderate associations or conducting subgroup analyses, and the risk of model overfitting cannot be excluded. (2) Single-population origin – All participants were recruited from a single population in Southwest China, which may restrict the generalizability of the findings to other ethnic or geographic groups. (3) Short follow-up – The relatively short follow-up period limited assessment of the long-term prognostic value of these biomarkers, including their potential to predict progression from acute to chronic osteomyelitis. Therefore, our results should be interpreted with caution. Future multicenter studies with larger, more diverse cohorts and extended follow-up are needed to further validate and establish the clinical utility of these biomarkers.

## Data Availability

The original contributions presented in the study are included in the article/Supplementary Material, further inquiries can be directed to the corresponding authors.
